# Whole-colon investigation vs. flexible sigmoidoscopy for suspected colorectal cancer based on presenting symptoms and signs: a multicentre cohort study

**DOI:** 10.1038/s41416-018-0335-z

**Published:** 2018-12-19

**Authors:** Amanda J. Cross, Kate Wooldrage, Emma C. Robbins, Kevin Pack, Jeremy P. Brown, William Hamilton, Michael R. Thompson, Karen G. Flashman, Steve Halligan, Siwan Thomas-Gibson, Margaret Vance, Brian P. Saunders, Wendy Atkin

**Affiliations:** 1 Cancer Screening and Prevention Research Group, Department of Surgery and Cancer, Imperial College London, London, UK; 2 Institute of Health Research, University of Exeter Medical School, Exeter, UK; 3 Department of Colorectal Surgery, Queen Alexandra Hospital, Portsmouth Hospitals NHS Trust, Portsmouth, UK; 4 University College London Centre for Medical Imaging, University College London, London, UK; 5 Wolfson Unit for Endoscopy, St Mark’s Hospital, London, UK

**Keywords:** Colorectal cancer, Physical examination, Digestive signs and symptoms, Anaemia, Endoscopy

## Abstract

**Background:**

Patients with suspected colorectal cancer (CRC) usually undergo colonoscopy. Flexible sigmoidoscopy (FS) may be preferred if proximal cancer risk is low. We investigated which patients could undergo FS alone.

**Methods:**

Cohort study of 7375 patients (≥55 years) referred with suspected CRC to 21 English hospitals (2004–2007), followed using hospital records and cancer registries. We calculated yields and number of needed whole-colon examinations (NNE) to diagnose one cancer by symptoms/signs and subsite. We considered narrow (haemoglobin <11 g/dL men; <10 g/dL women) and broad (<13 g/dL men; <12 g/dL women) anaemia definitions and iron-deficiency anaemia (IDA).

**Results:**

One hundred and twenty-seven proximal and 429 distal CRCs were diagnosed. A broad anaemia definition identified 80% of proximal cancers; a narrow definition with IDA identified 39%. In patients with broad definition anaemia and/or abdominal mass, proximal cancer yield and NNE were 4.8% (97/2022) and 21. In patients without broad definition anaemia and/or abdominal mass, with rectal bleeding or increased stool frequency (41% of cohort), proximal cancer yield and NNE were 0.4% (13/3031) and 234.

**Conclusion:**

Most proximal cancers are accompanied by broad definition anaemia. In patients without broad definition anaemia and/or abdominal mass, with rectal bleeding or increased stool frequency, proximal cancer is rare and FS should suffice.

## Introduction

Patients referred to hospital with suspected colorectal cancer (CRC) typically undergo whole-colon investigation (WCI), predominantly colonoscopy or computed tomography (CT) colonography, in line with National Institute for Health and Care Excellence (NICE) guidelines.^1^ In 2015, NICE issued a guideline on referral criteria, including symptoms and signs conferring a positive predictive value for cancer of 3%.^2^ Consequently, large numbers of patients are undergoing WCI for suspected CRC, placing pressure on endoscopy and radiology services and incurring substantial costs to the National Health Service (NHS).^3,4^ Reducing the burden of symptomatic referrals on diagnostic services is recognised as a priority area for research.^5^

Flexible sigmoidoscopy is quicker, safer, less complicated, and cheaper than colonoscopy. Intravenous sedation is usually not needed and enemas used for preparation are associated with fewer side effects and greater acceptability than oral preparations used for WCI.^6^ Flexible sigmoidoscopy can be performed competently by non-physician endoscopists^7^ and has high sensitivity for CRCs in the distal colon and rectum^8–10^; however, it can only reach the splenic flexure at best and so abnormalities in the proximal colon are only found if distal findings precipitate subsequent WCI.

Previous research has demonstrated that presenting symptoms/signs are associated with CRC location.^8–14^ In the largest previous study, Thompson et al. defined a patient subgroup at low risk of proximal cancer, for whom they deemed examination by flexible sigmoidoscopy alone appropriate.^8^ However, subsequent studies reached variable conclusions regarding sole use of flexible sigmoidoscopy in any patient subgroup,^9–14^ and the current NICE guidelines only recommend it for patients with major comorbidity, in association with barium enema.^1^ The aim of the present SOCCER (Symptoms of Colorectal Cancer Evaluation Research) study^15^ was to further investigate whether presenting symptoms/signs could be used to identify patients at low risk of proximal cancer, for whom flexible sigmoidoscopy would suffice. This would help alleviate the burden of WCI on patients and endoscopy and radiology services.

## Methods

### Study design and participants

We included patients who had been referred to 1 of the 21 English NHS hospitals from 2004 to 2007 for investigation of symptoms/signs suggestive of CRC. Of these hospitals, 8 were general and 13 were teaching hospitals, and they varied from <40 to >1000 beds in size.

We retrospectively identified patients from those who were assessed for eligibility for the SIGGAR (Special Interest Group in Gastrointestinal and Abdominal Radiology) trials. The SIGGAR trials were two parallel randomised controlled trials assessing the clinical and cost-effectiveness of CT colonography vs. barium enema and colonoscopy for CRC diagnosis.^16,17^ To be eligible for the SIGGAR trials, patients had to be ≥55 years and judged to be in need of, and fit enough for, a WCI with full bowel preparation. Patients were ineligible if they were in follow-up for CRC, had undergone WCI within the previous 6 months, had a known diagnosis of familial adenomatous polyposis or Lynch syndrome, or had previously been diagnosed with inflammatory bowel disease (IBD).

Research nurses undertook the eligibility assessment, checking endoscopy and radiology databases, and patient records and notes. Only patients who were deemed eligible, gave informed consent, and had a consultant consent to their participation were randomised in the SIGGAR trials. Reasons for patient- and consultant-declined consent included patients wanting to have or avoid a specific type of WCI, consultants requesting a specific procedure, and prior cancer diagnoses.

**Fig. 1:**
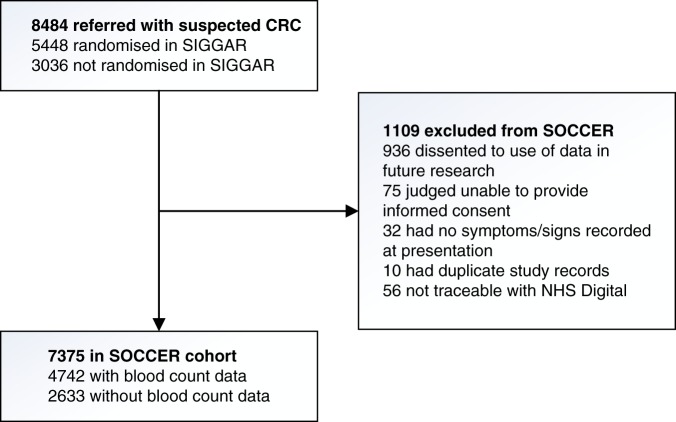
Flow chart of study participants CRC colorectal cancer, SIGGAR Special Interest Group in Gastrointestinal and Abdominal Radiology, SOCCER Symptoms of Colorectal Cancer Evaluation Research. The SOCCER study included patients who had been referred to hospital with suspected CRC and assessed for eligibility for the SIGGAR trials. All patients who met the SIGGAR trials eligibility criteria (regardless of whether they proceeded to randomisation) were eligible for the SOCCER study, unless they had dissented to use of their data in future research, were judged unable by a clinician to provide informed consent for the use of their data in future research, had no symptoms/signs recorded at presentation, had duplicate study records, or were not traceable with NHS Digital. Among those included in the SOCCER study, data on haemoglobin and mean red cell corpuscular volume were available for 4742 of the 7375 patients

All patients who met the SIGGAR trial eligibility criteria (regardless of whether they proceeded to randomisation) were eligible for the SOCCER study, unless they were judged incapable of giving informed consent, had dissented to use of their data for research, had no symptoms/signs documented at presentation, had duplicate study records, or were untraceable through NHS Digital (Fig. 1).

### Data collection and management

Research nurses or colorectal administrative assistants examined patient records and notes, including referral letters, and recorded demographic and clinical details on bespoke SIGGAR trial pro-formas, including age, sex, general practitioner (GP)-reported symptoms, route and urgency of referral, and planned diagnostic investigations. There were tick boxes for ‘abdominal pain’, ‘anaemia’, ‘change in bowel habit (CIBH)’, ‘positive FOBT’ (faecal occult blood test), ‘rectal bleeding’, and ‘weight loss’. Free text fields were included to record additional symptoms/signs that were coded and classified, and characteristics of reported CIBHs, classified as ‘more frequent’, ‘less frequent’, ‘variable’, or ‘unspecified’. Blood count data, additional clinical features, diagnostic investigations performed, and diagnoses during the hospital episode were ascertained through hospital record review.

For patients with blood count data, anaemia status was determined according to the results of blood tests performed within 6 months before and up to 3 months after referral. We examined the prevalence of anaemia and iron-deficiency anaemia (IDA) using two anaemia definitions: haemoglobin (Hb) <13 g/dL in men and <12 g/dL in women (broad definition anaemia), used by the World Health Organisation; and Hb < 11 g/dL in men and <10 g/dL in women (narrow definition anaemia), as in the 2005 NICE referral guidelines.^18,19^ IDA was classified on the basis of microcytosis (mean red cell corpuscular volume [MCV] <80 fL) or serum ferritin <20 μg/L. For patients without blood count data, we defined anaemia based on whether the investigation of anaemia was indicated as a reason for referral on the pro-forma.

CRC diagnoses occurring within 3 years of referral were obtained from cancer registries, via NHS Digital, and hospital records. CRC sites were defined by International Classification of Diseases, tenth revision codes: proximal cancer included C18.0–C18.5 (caecum to splenic flexure) and distal cancer included C18.6, C18.7, C19, C20, and C21 (descending colon to anus). CRC morphologies were defined by International Classification of Diseases for Oncology, second edition codes; we included codes related to invasive and in situ carcinomas (8000/3, 8010/3, 8070/3, 8123/3, 8140/2, 8140/3, 8144/3, 8210/3, 8261/2, 8261/3, 8263/2, 8263/3, 8480/3, 8481/3, 8490/3, 8510/3, and 8560/3).

### Statistical analysis

The primary outcome was the yield of proximal vs. distal cancer within 3 years of referral by presenting symptom/sign. Yields were calculated as the number of patients with proximal or distal cancer divided by the number of patients with a particular symptom/sign, presented as percentages. Secondary outcomes included the number of needed whole-colon examinations (NNE) to detect one proximal vs. distal cancer by presenting symptom/sign, calculated by inverting the yield and presented with binomial exact 95% confidence intervals (CIs). For this calculation, we made the assumption that WCI has perfect sensitivity for detecting proximal and distal cancers.

We calculated the sensitivity of symptoms/signs for proximal and distal cancer and the proportion of patients with CRC who had proximal vs. distal cancer by presenting symptom/sign. We also calculated the proportion of CRCs that would have been missed if certain symptoms/signs were investigated by flexible sigmoidoscopy alone, with binomial exact 95% CIs, assuming that flexible sigmoidoscopy would have detected all distal cancers but not proximal cancers.

We estimated that, with a sample size of 8,484 patients, giving rise to an estimated 68 proximal and 421 distal cancers, we could estimate with sufficient precision the proportion of CRCs that would have been missed if certain symptoms/signs were investigated by flexible sigmoidoscopy alone. Data analyses were conducted using Stata/IC 13.1 (StataCorp. 2013. Stata Statistical Software: Release 13. College Station, TX: StataCorp LP).

## Results

A total of 8484 patients referred to hospital with suspected CRC were assessed for eligibility for the SIGGAR trials. Of these, 5448 were randomised to the SIGGAR trials and 3036 were not (Fig. 1). Comparing randomised and non-randomised patients, the former were younger and less likely to have been referred from a colorectal surgical outpatient clinic and via the urgent pathway (data not shown).

Of the total 8484 patients, all of whom were deemed eligible for the SOCCER study, 1109 were subsequently excluded, primarily due to patient dissent to use of their data for research. This left 7375 patients for our cohort analysis (Fig. 1). The median age of included patients was 69 years (interquartile range: 62–76) and 59.0% were women. The majority of patients were referred from a colorectal surgical outpatient clinic (84.5%) and via the urgent pathway (71.7%) (Table 1), and 1483 (20.1%) had undergone flexible sigmoidoscopy at the time of referral (data not shown).

**Table 1:** Patient characteristics (*N* = 7375)

Characteristic	*N*	%
Sex		
Men	3023	41.0
Women	4352	59.0
Age (years)		
55–64	2407	32.6
65–74	2739	37.1
75–84	1896	25.7
≥85	333	4.5
Route of referral		
Colorectal surgical outpatient clinic	6231	84.5
Other outpatient clinic	688	9.3
Straight to test	396	5.4
Hospital admission	33	0.4
Not recorded	27	0.4
Urgency of referral		
Urgent	5290	71.7
Soon	660	9.0
Routine	914	12.4
Not recorded	511	6.9
Availability of blood count data^a^		
Hb and MCV	4742	64.3
Serum ferritin	1157	15.7
None available	2633	35.7

^a^All patients with serum ferritin had a haemoglobin (Hb) and mean red cell corpuscular volume (MCV) count

In total, 556 CRCs were diagnosed in 551 of the 7375 patients (7.5%) within 3 years following referral (Table 2). Of the 551 patients with CRC, 522 (94.7%) were diagnosed within 6 months post-referral (data not shown). There were 127 proximal and 429 distal cancers (5 patients had synchronous proximal and distal cancer), giving diagnostic yields of 1.7% for proximal cancer and 5.8% for distal cancer (Table 2). Detailed subsite information is presented in Supplementary Table 1.

**Table 2:** Yield of distal and proximal cancer overall and according to anaemia status (*N* = 7375)

Group	Anaemia status	All referred patients		Distal cancer^a^		Proximal cancer^a^		
		*n*	% of group	Patients with distal cancer, *n*	Diagnostic yield, %	Patients with proximal cancer, *n*	Diagnostic yield, %	Proportion of all proximal cancers in the group, %
All patients		7375	100	429	5.8	127	1.7	100
Patients with blood count data available	Any	4742	100	240	5.1	97	2.0	100
	Anaemia: narrow definition							
	Hb < 11 g/dL in men and <10 g/dL in women							
	Total	672	14.2	38	5.7	51	7.6	52.6 (51/97)
	With IDA^b^	363	7.7	22	6.1	38	10.5	39.2 (38/97)
	Without IDA	309	6.5	16	5.2	13	4.2	13.4 (13/97)
	Anaemia: broad definition (WHO)							
	Hb < 13 g/dL in men and <12 g/dL in women							
	Total	1660	35.0	106	6.4	78	4.7	80.4 (78/97)
	With IDA^b^	567	12.0	36	6.3	48	8.5	49.5 (48/97)
	Without IDA	1093	23.0	70	6.4	30	2.7	30.9 (30/97)
	No anaemia							
	Hb ≥ 13 g/dL in men and ≥12 g/dL in women							
	Total	3082	65.0	134	4.3	19	0.6	19.6 (19/97)
	With IDA^b^	66	1.4	2	3.0	2	3.0	2.1 (2/97)
	Without IDA	3016	63.6	132	4.4	17	0.6	17.5 (17/97)
Patients with no blood count data available	Any	2633	100	189	7.2	30	1.1	100
	Anaemia indicated as a reason for referral	229	8.7	19	8.3	9	3.9	30.0 (9/30)
	Anaemia not indicated as a reason for referral	2404	91.3	170	7.1	21	0.9	70.0 (21/30)
All patients (with and without blood count data)	Anaemia (by definition used in paper^c^)	1889	25.6	125	6.6	87	4.6	68.5 (87/127)
	No anaemia^c^	5486	74.4	304	5.5	40	0.7	31.5 (40/127)

^a^Five patients had both a distal and proximal cancer diagnosed. These patients are included in both the distal and proximal cancer columns

^b^Iron-deficiency anaemia (IDA) was classified on the basis of microcytosis [mean red cell corpuscular volume (MCV) <80 fL] or serum ferritin <20 μg/L. We did not have serum ferritin measurements for all patients with haemoglobin (Hb) counts

^c^In patients with blood count data, anaemia was defined by the WHO (broad) definition (Hb level <13 g/dL in men or <12 g/dL in women). In patients without blood count data, anaemia was defined by whether the investigation of anaemia was indicated as a reason for referral

Blood count data were not available for all patients. Data on Hb and MCV were available for 4742 of 7375 patients (64.3%). There were 1157 patients (15.7%) with serum ferritin in addition to Hb and MCV counts (Table 1). Comparing the 4742 patients with Hb and MCV data to the 2633 patients without, those with data available were older and less likely to have been referred from a colorectal outpatient clinic (Supplementary Table 2).

Among the 4742 patients with blood count data, narrow definition anaemia was present in 672 (14.2%) and narrow definition anaemia with IDA was present in 363 (7.7%). Broad definition anaemia was present in 1660 (35.0%) and broad definition anaemia with IDA in 567 (12.0%) (Table 2). The prevalence of anaemia increased with age among men and women. Broad definition anaemia was present in 23.8% (131/551) of men and in 18.6% (161/867) of women aged 55–64 years and in 73.3% (66/90) of men and 57.2% (83/145) of women aged ≥85 years. This pattern of anaemia increasing with age was also observed when considering the other anaemia definitions and IDA (data not shown). Among the 2633 patients without blood count data, investigation of anaemia was a reason for referral in 229 (8.7%) (Table 2).

### Anaemia and cancer site

Among the 4742 patients with blood count data, there were 97 proximal and 240 distal cancers. While yields of distal cancer did not vary by anaemia status, anaemia was strongly associated with proximal cancer (Table 2). Proximal cancer yield was 0.6% (19/3082) in patients without anaemia, increasing to 10.5% (38/363) in patients with narrow definition anaemia and IDA. Broadening the definition of anaemia and removing the requirement for IDA reduced the yield to 4.7% (78/1660). Among the 2633 patients without blood count data, yield of proximal cancer was 3.9% (9/229) in patients referred for investigation of anaemia vs. 0.9% (21/2404) in those who were not (Table 2).

Although the yield of proximal cancer was highest in patients with narrow definition anaemia and IDA, this criterion identified 39.2% (38/97) of patients with proximal cancer, while the broad definition of anaemia (with or without IDA) identified 80.4% (78/97) (Table 2). We therefore used the broad definition in all subsequent analyses of patients with blood count data (*n* = 4742) to ensure high sensitivity of anaemia for proximal cancer. For patients without blood count data (*n* = 2633), we defined anaemia based on whether the investigation of anaemia was indicated as a reason for referral. Among all 7375 patients, 1889 (25.6%) had either broad definition anaemia (*n* = 1660) or were referred for investigation of anaemia (*n* = 229) (Table 2).

### Symptoms and signs related to cancer site

Among all patients, the most common symptoms/signs were a CIBH (73.0%, *n* = 5382), rectal bleeding (37.6%, *n* = 2773), abdominal pain (28.8%, *n* = 2126), weight loss (15.6%, *n* = 1148), and anaemia (25.6%, *n* = 1889) (Table 3). Most patients presented with more than one symptom/sign (Supplementary Table 3).

**Table 3:** Yield of distal and proximal cancer by combinations of symptoms and signs (*N* = 7375)

	Total patients						Additional NICE 2015 guideline symptoms or signs^a^																																							
							None					Change in bowel habit					Rectal bleeding					Abdominal pain					Weight loss					Anaemia					Abdominal mass					Rectal mass				
	Cancers						Cancers					Cancers					Cancers					Cancers					Cancers					Cancers					Cancers					Cancers				
	Total		Distal		Proximal		Total	Distal		Proximal		Total	Distal		Proximal		Total	Distal		Proximal		Total	Distal		Proximal		Total	Distal		Proximal		Total	Distal		Proximal		Total	Distal		Proximal		Total	Distal		Proximal	
	*n*	%	*n*	%	*n*	%	*n*	*n*	%	*n*	%	*n*	*n*	%	*n*	%	*n*	*n*	%	*n*	%	*n*	*n*	%	*n*	%	*n*	*n*	%	*n*	%	*n*	*n*	%	*n*	%	*n*	*n*	%	*n*	%	*n*	*n*	%	*n*	%
Symptoms																																														
Change in bowel habit	5382	73.0	308	5.7	70	1.3	1589	33	2.1	7	0.4	—	—	—	—	—	1674	196	11.7	18	1.1	1602	79	4.9	29	1.8	920	62	6.7	18	2.0	1085	77	7.1	39	3.6	134	13	9.7	10	7.5	97	34	35.1	0	0
More frequent	2862	38.8	159	5.6	36	1.3	908	17	1.9	2	0.2	—	—	—	—	—	811	100	12.3	13	1.6	811	35	4.3	15	1.8	525	40	7.6	7	1.3	565	45	8.0	18	3.2	63	4	6.3	4	6.3	36	10	27.8	0	0
Less frequent	865	11.7	43	5.0	13	1.5	189	5	2.6	2	1.1	—	—	—	—	—	307	26	8.5	2	0.7	277	12	4.3	6	2.2	171	6	3.5	4	2.3	211	12	5.7	8	3.8	32	3	9.4	1	3.1	17	6	35.3	0	0
Variable	648	8.8	26	4.0	8	1.2	183	8	4.4	1	0.5	—	—	—	—	—	188	8	4.3	1	0.5	235	7	3.0	4	1.7	103	2	1.9	5	4.9	113	8	7.1	4	3.5	11	2	18.2	2	18.2	9	2	22.2	0	0
Unspecified	1007	13.7	80	7.9	13	1.3	309	3	1.0	2	0.6	—	—	—	—	—	368	62	16.8	2	0.5	279	25	9.0	4	1.4	121	14	11.6	2	1.7	196	12	6.1	9	4.6	28	4	14.3	3	10.7	35	16	45.7	0	0
Rectal bleeding	2773	37.6	291	10.5	30	1.1	618	47	7.6	3	0.5	1674	196	11.7	18	1.1	—	—	—	—	—	622	57	9.2	6	1.0	300	37	12.3	5	1.7	549	66	12.0	16	2.9	48	7	14.6	3	6.3	95	43	45.3	1	1.1
Abdominal pain	2126	28.8	96	4.5	42	2.0	192	2	1.0	0	0	1602	79	4.9	29	1.8	622	57	9.2	6	1.0	—	—	—	—	—	355	26	7.3	12	3.4	384	24	6.3	25	6.5	93	8	8.6	13	14.0	26	9	34.6	0	0
Weight loss	1148	15.6	77	6.7	28	2.4	18	0	0	0	0	920	62	6.7	18	2.0	300	37	12.3	5	1.7	355	26	7.3	12	3.4	—	—	—	—	—	425	35	8.2	22	5.2	58	5	8.6	5	8.6	13	1	7.7	0	0
Other symptoms^b^	479	6.5	20	4.2	10	2.1	—	—	—	—	—	373	15	4.0	9	2.4	127	11	8.7	1	0.8	177	5	2.8	2	1.1	140	8	5.7	3	2.1	117	8	6.8	7	6.0	23	3	13.0	1	4.3	11	1	9.1	0	0
Signs																																														
Anaemia^c^	1889	25.6	125	6.6	87	4.6	404	13	3.2	24	5.9	1085	77	7.1	39	3.6	549	66	12.0	16	2.9	384	24	6.3	25	6.5	425	35	8.2	22	5.2	—	—	—	—	—	83	8	9.6	10	12.0	31	4	12.9	1	3.2
Abdominal mass	216	2.9	19	8.8	20	9.3	12	1	8.3	4	33.3	134	13	9.7	10	7.5	48	7	14.6	3	6.3	93	8	8.6	13	14.0	58	5	8.6	5	8.6	83	8	9.6	10	12.0	—	—	—	—	—	3	2	66.7	0	0
Rectal mass	165	2.2	47	28.5	1	0.6	19	1	5.3	0	0	97	34	35.1	0	0	95	43	45.3	1	1.1	26	9	34.6	0	0	13	1	7.7	0	0	31	4	12.9	1	3.2	3	2	66.7	0	0	—	—	—	—	—
Other signs^d^	265	3.6	13	4.9	5	1.9	—	—	—	—	—	153	8	5.2	2	1.3	77	6	7.8	0	0	68	4	5.9	3	4.4	34	4	11.8	0	0	72	6	8.3	4	5.6	2	0	0	0	0	4	0	0	0	0

Percentages for distal and proximal cancers are yields. Five patients had both a distal and proximal cancer diagnosed. These patients are included in both the distal and proximal cancer columns. The symptoms occurring in these patients were: anaemia and rectal bleeding; anaemia, change in bowel habit (CIBH) (more frequent) and weight loss; rectal bleeding, CIBH (more frequent), abdominal pain and weight loss; CIBH (more frequent) and bloating; and CIBH (unspecified) alone

^a^NICE 2015 guideline symptoms/signs included CIBH, rectal bleeding, abdominal pain, weight loss, anaemia and abdominal or rectal mass without any restriction by age; the broad definition of anaemia was used. Patients may have had multiple symptoms/signs

^b^Other symptoms include bloating/flatulence (*n* = 203), tiredness/weakness (*n* = 152), anal symptoms (*n* = 97), nausea/vomiting (*n* = 44), back pain (*n* = 13), and upper gastrointestinal symptoms (*n* = 10)

^c^In patients with blood count data, anaemia was defined by the WHO (broad) definition [haemoglobin (Hb) level <13 g/dL in men or <12 g/dL in women]. In patients without blood count data, anaemia was defined by whether the investigation of anaemia was indicated as a reason for referral

^d^Other signs include faecal occult blood test (FOBT) positivity (*n* = 113), family history (*n* = 117), history of polyps (*n* = 23), cancer antibodies (*n* = 3), elevated C-reactive protein (*n* = 4), and liver problems (*n* = 9)

Yields of proximal and distal cancer varied considerably by symptom/sign (Table 3). The highest yields of distal cancer were among patients with rectal mass (28.5%, 47/165) and rectal bleeding (10.5%, 291/2773). Yields of proximal cancer were generally lower than for distal cancer, with the highest yields among patients with abdominal mass (9.3%, 20/216) and anaemia (4.6%, 87/1889). Yields of proximal and distal cancer were generally higher when patients presented with a combination of symptoms/signs (Table 3).

The location of diagnosed cancers was also influenced by symptom/sign (Table 4). Rectal bleeding was associated with an approximate 90% chance that cancer was distal, irrespective of additional symptoms. Anaemia and abdominal mass were associated with a high probability that cancer was proximal (41.4% and 51.3%, respectively), irrespective of additional symptoms.

**Table 4:** Proportion of all cancers located distally and proximally by combinations of symptoms and signs (*N* = 7375)

	Total patients						Additional NICE 2015 guideline symptoms or signs^a^																															
							None				Change in bowel habit				Rectal bleeding				Abdominal pain				Weight loss				Anaemia^b^				Abdominal mass				Rectal mass			
	Cancers						Cancers				Cancers				Cancers				Cancers				Cancers				Cancers				Cancers				Cancers			
	Total		Distal		Proximal		Distal		Proximal		Distal		Proximal		Distal		Proximal		Distal		Proximal		Distal		Proximal		Distal		Proximal		Distal		Proximal		Distal		Proximal	
	*n*	%	*n*	%	*n*	%	*n*	%	*n*	%	*n*	%	*n*	%	*n*	%	*n*	%	*n*	%	*n*	%	*n*	%	*n*	%	*n*	%	*n*	%	*n*	%	*n*	%	*n*	%	*n*	%
Symptoms																																						
Change in bowel habit	5382	73.0	308	82.3	70	18.7	33	86.8	7	18.4	—	—	—	—	196	92.0	18	8.5	79	73.8	29	27.1	62	79.4	18	23.1	77	67.0	39	33.9	13	56.5	10	43.5	34	100	0	0
More frequent	2862	38.8	159	82.8	36	18.8	17	94.4	2	11.1	—	—	—	—	100	89.3	13	11.6	35	71.4	15	30.6	40	88.9	7	15.6	45	72.6	18	29.0	4	50.0	4	50.0	10	100	0	0
Less frequent	865	11.7	43	76.8	13	23.2	5	71.4	2	28.6	—	—	—	—	26	92.9	2	7.1	12	66.7	6	33.3	6	60.0	4	40.0	12	60.0	8	40.0	3	75.0	1	25.0	6	100	0	0
Variable	648	8.8	26	76.5	8	23.5	8	88.9	1	11.1	—	—	—	—	8	88.9	1	11.1	7	63.4	4	36.4	2	28.6	5	71.4	8	66.7	4	33.3	2	50.0	2	50.0	2	100	0	0
Unspecified	1007	13.7	80	87.0	13	14.1	3	75.0	2	50.0	—	—	—	—	62	96.9	2	3.1	25	86.2	4	13.8	14	87.5	2	12.5	12	57.1	9	42.9	4	57.1	3	42.9	16	100	0	0
Rectal bleeding	2773	37.6	291	91.2	30	9.4	47	94.0	3	6.0	196	92.0	18	8.5	—	—	—	—	57	91.9	6	9.7	37	90.2	5	12.2	66	81.5	16	19.8	7	70.0	3	30.0	43	97.7	1	2.3
Abdominal pain	2126	28.8	96	70.1	42	30.7	2	100	0	0	79	73.8	29	27.1	57	91.9	6	9.7	—	—	—	—	26	70.3	12	32.4	24	49.0	25	51.0	8	38.1	13	61.9	9	100	0	0
Weight loss	1148	15.6	77	74.8	28	27.2	0	0	0	0	62	79.5	18	23.1	37	90.2	5	12.2	26	70.3	12	32.4	—	—	—	—	35	62.5	22	39.3	5	50.0	5	50.0	1	100	0	0
Other symptoms^b^	479	6.5	20	69.0	10	34.5	—	—	—	—	15	65.2	9	39.1	11	91.7	1	8.3	5	71.4	2	28.6	8	72.7	3	27.3	8	53.3	7	46.7	3	75.0	1	25.0	1	100	0	0
Signs																																						
Anaemia^c^	1889	25.6	125	59.5	87	41.4	13	35.1	24	64.9	77	67.0	39	33.9	66	81.5	16	19.8	24	49.0	25	51.0	35	62.5	22	39.3	—	—	—	—	8	44.4	10	55.6	4	80.0	1	20.0
Abdominal mass	216	2.9	19	48.7	20	51.3	1	20.0	4	80.0	13	56.5	10	43.5	7	70.0	3	30.0	8	38.1	13	61.9	5	50.0	5	50.0	8	44.4	10	55.6	—	—	—	—	2	100	0	0
Rectal mass	165	2.2	47	97.9	1	2.1	1	100	0	0	34	100	0	0	43	97.7	1	2.3	9	100	0	0	1	100	0	0	4	80.0	1	20.0	2	100	0	0	—	—	—	—
Other signs^d^	265	3.6	13	72.2	5	27.8	—	—	—	—	8	80.0	2	20.0	6	100	0	0	4	57.1	3	42.9	4	100	0	0	6	60.0	4	40.0	0	0	0	0	0	0	0	0

Percentages are the proportion of all cancers that are located in that site. Five patients had both a distal and proximal cancer diagnosed. These patients are included in both the distal and proximal cancer columns. The symptoms occurring in these patients were: anaemia and rectal bleeding; anaemia, change in bowel habit (CIBH) (more frequent) and weight loss; rectal bleeding, CIBH (more frequent), abdominal pain and weight loss; CIBH (more frequent) and bloating; and CIBH (unspecified) alone

^a^NICE 2015 guideline symptoms/signs included CIBH, rectal bleeding, abdominal pain, weight loss, anaemia and abdominal or rectal mass without any restriction by age; the broad definition of anaemia was used. Patients may have had multiple symptoms/signs

^b^Other symptoms include bloating/flatulence (*n* = 203), tiredness/weakness (*n* = 152), anal symptoms (*n* = 97), nausea/vomiting (*n* = 44), back pain (*n* = 13), and upper gastrointestinal symptoms (*n* = 10)

^c^In patients with blood count data, anaemia was defined by the WHO (broad) definition [haemoglobin (Hb) level <13 g/dL in men or <12 g/dL in women]. In patients without blood count data, anaemia was defined by whether the investigation of anaemia was indicated as a reason for referral

^d^Other signs include faecal occult blood test (FOBT) positivity (*n* = 113), family history (*n* = 117), history of polyps (*n* = 23), cancer antibodies (*n* = 3), elevated C-reactive protein (*n* = 4), and liver problems (*n* = 9)

We evaluated the combination of anaemia and/or abdominal mass in further detail, given the strong association with proximal cancer. Among the 2022 patients with anaemia and/or abdominal mass, proximal cancer yield was 4.8%, the NNE was 21 (95% CI 18–26), and 42.0% of the patients with cancer had proximal cancer. The sensitivity of anaemia and/or abdominal mass for proximal cancer was 76.4% (97/127) (Table 5).

**Table 5:** Symptom combination at presentation and yield of distal and proximal cancer (*N* = 7375)

Symptom/signs combinations	Total patients		Distal cancers^a^				Proximal cancers^a^				All sites		
	*n*	%	Patients with distal cancer, *n*	Proportion of distal cancers, %	Diagnostic yield, %	No. needed to examine to diagnose one cancer (95% CI)	Patients with proximal cancer, *n*	Proportion of proximal cancers, %	Diagnostic yield, %	No. needed to examine to diagnose one cancer (95% CI)	Patients with cancer, *n*	Proportion with a distal cancer, %	Proportion with a proximal cancer, %
Total	7375	100	429	100.0	5.8	18 (16, 19)	127	100.0	1.7	59 (49, 70)	551	77.9	23.0
Anaemia^b^ and/or abdominal mass	2022	27.4	136	31.7	6.7	15 (13, 18)	97	76.4	4.8	21 (18, 26)	231	58.9	42.0
Anaemia^b^, no abdominal mass	1806	24.5	117	27.3	6.5	16 (13, 19)	77	60.6	4.3	24 (19, 30)	192	60.9	40.1
Abdominal mass, no anaemia^b^	133	1.8	11	2.6	8.3	13 (7, 24)	10	7.9	7.5	14 (8, 28)	21	52.4	47.6
Anaemia^b^ and abdominal mass	83	1.1	8	1.9	9.6	11 (6, 24)	10	7.9	12.0	9 (5, 17)	18	44.4	55.6
No anaemia^b^ or abdominal mass													
Total	5353	72.6	293	68.3	5.5	19 (17, 21)	30	23.6	0.6	179 (126, 265)	320	91.6	9.4
Rectal bleeding													
Total	2195	29.8	221	51.5	10.1	10 (9, 12)	13	10.2	0.6	169 (99, 317)	233	94.8	5.6
Rectal bleeding alone	571	7.7	43	10.0	7.5	14 (10, 19)	3	2.4	0.5	191 (66, 922)	46	93.5	6.5
Rectal bleeding and CIBH	1345	18.2	156	36.4	11.6	9 (8, 11)	10	7.9	0.7	135 (74, 281)	165	94.5	6.1
Rectal bleeding and weight loss or abdominal pain, no CIBH	202	2.7	8	1.9	4.0	26 (14, 58)	0	0.0	0	—	8	100	0.0
Rectal bleeding and only other symptoms/signs^c^	77	1.0	14	3.3	18.2	6 (4, 10)	0	0.0	0	—	14	100	0.0
CIBH, no rectal bleeding													
Total	2866	38.9	68	15.9	2.4	43 (34, 55)	17	13.4	0.6	169 (106, 290)	83	81.9	20.5
CIBH alone	1464	19.9	31	7.2	2.1	48 (34, 70)	5	3.9	0.3	293 (126, 902)	35	88.6	14.3
More frequent	836	11.3	15	3.5	1.8	56 (34, 100)	0	0.0	0	—	15	100	0.0
Less frequent	174	2.4	5	1.2	2.9	35 (16, 107)	2	1.6	1.1	87 (25, 717)	7	71.4	28.6
Variable	166	2.3	8	1.9	4.8	21 (11, 48)	1	0.8	0.6	166 (31, 6558)	9	88.9	11.1
Unspecified	288	3.9	3	0.7	1.0	96 (34, 465)	2	1.6	0.7	144 (41, 1188)	4	75.0	50.0
CIBH and weight loss or abdominal pain	1253	17.0	34	7.9	2.7	37 (27, 54)	10	7.9	0.8	126 (69, 261)	44	77.3	22.7
CIBH and only other symptoms/signs^c^	149	2.0	3	0.7	2.0	50 (18, 240)	2	1.6	1.3	75 (21, 614)	4	75.0	50.0
No rectal bleeding or CIBH													
Abdominal pain or weight loss	241	3.3	2	0.5	0.8	121 (34, 994)	0	0.0	0	—	2	100	0.0
Only other symptoms/signs^c^	51	0.7	2	0.5	3.9	26 (8, 209)	0	0.0	0	—	2	100	0.0
Suggested criteria for flexible sigmoidoscopy													
No anaemia or abdominal mass													
Rectal bleeding	2195	29.8	221	51.5	10.1	10 (9, 12)	13	10.2	0.6	169 (99, 317)	233	94.8	5.6
CIBH to more frequent alone	836	11.3	15	3.5	1.8	56 (34, 100)	0	0.0	0	—	15	100	0.0
Total	3031	41.1	236	55.0	7.8	13 (12, 15)	13	10.2	0.4	234 (137, 438)	248	95.2	5.2

^a^Five patients had both a distal and proximal cancer diagnosed. These patients are included in both the distal and proximal cancer columns. Of these five patients, two had anaemia, one had rectal bleeding and a change in bowel habit (CIBH) (more frequent), one had a CIBH (more frequent) and bloating, and one had a CIBH (unspecified) alone

^b^In patients with blood count data, anaemia was defined by the WHO (broad) definition [haemoglobin (Hb) level <13 g/dL in men or <12 g/dL in women]. In patients without blood count data, anaemia was defined by whether the investigation of anaemia was indicated as a reason for referral

^c^Includes bloating/flatulence (*n* = 203), tiredness/weakness (*n* = 152), anal symptoms (*n* = 97), nausea/vomiting (*n* = 44), back pain (*n* = 13), upper gastrointestinal symptoms (*n* = 10), faecal occult blood test (FOBT) positivity (*n* = 113), family history (*n* = 117), history of polyps (*n* = 23), cancer antibodies (*n* = 3), elevated C-reactive protein (*n* = 4), and liver problems (*n* = 9)

Among the 5353 patients without anaemia and/or abdominal mass, the yield of proximal cancer was 0.6% (NNE 179, 95% CI 126–265). Proximal cancer yield was also 0.6% (NNE 169, 95% CI 99–317) among the 2195 patients without anaemia and/or abdominal mass who had rectal bleeding. No proximal cancers were found in 836 patients without anaemia and/or abdominal mass who presented with a CIBH to increased frequency alone (Table 5).

There were 3031 patients without anaemia and/or abdominal mass who had rectal bleeding or solely a CIBH to increased frequency, accounting for 41.1% of the cohort. Among these patients, 236 distal cancers were diagnosed (yield of 7.8%; NNE 13, 95% CI 12–15) while only 13 proximal cancers were diagnosed (yield of 0.4%; NNE 234, 95% CI 137–438) (Table 5). Yields of proximal cancer were <1% for all age ranges in this patient subgroup (data not shown). Of the 13 proximal cancer patients, 6 had distal findings that would warrant WCI (Supplementary Table 4). Therefore, if this patient subgroup were investigated by flexible sigmoidoscopy alone, 7 of the total 556 cancers (1.3%, 95% CI 0.5–2.6%) would have been missed. For five of these seven cases, blood count data were not available and so anaemia at presentation cannot be ruled out.

Flexible sigmoidoscopy is considered complete if the sigmoid–descending colon junction is reached^20^; therefore, cancers in the descending colon may be missed. Descending colon cancers were rare in our study (1.4%, 8/556). Of the eight patients with descending colon cancers, two had the symptom profile of rectal bleeding or solely a CIBH to increased frequency without anaemia and/or abdominal mass; one of these had a synchronous cancer in the sigmoid colon while the other had no important distal findings (Supplementary Table 5).

## Discussion

For patients referred to hospital with suspected CRC, an important decision is whether to offer WCI or flexible sigmoidoscopy. NHS clinics offering rapid access flexible sigmoidoscopy have been shown to be suitable for evaluation of urgently referred patients, with a low miss rate of proximal cancer.^9,11^ However, fear of missing proximal cancers remains, with 20–70% of patients proceeding to WCI following flexible sigmoidoscopy.^8,9,11,21^ This negates the convenience and cost-effectiveness of flexible sigmoidoscopy, particularly as subsequent WCI has a very low cancer yield.^8,9^ To address the fear of missing cancers in patients examined solely by flexible sigmoidoscopy, we sought to determine in which patients the yield of proximal cancer and the probability of missing proximal cancer is low.

This multicentre cohort study of 7375 patients referred with suspected CRC to 21 hospitals throughout England validates previous studies that showed how yields of proximal and distal cancer vary by presenting symptom/sign.^8–14^ The largest previous study of 16,433 patients referred to hospital from 1986 to 2001 concluded that flexible sigmoidoscopy alone was sufficient for patients with a CIBH, rectal bleeding, and/or abdominal pain but without IDA, abdominal mass, severe symptoms, or significant flexible sigmoidoscopy findings. This was based on the finding that proximal cancer was very rare (<1%) in such patients.^8^ In an extension of this study, including data collected from 1986 to 2007, proximal cancer was again associated with IDA and abdominal mass but not with a CIBH or rectal bleeding.^22^

Our study confirms the strong association between anaemia and abdominal mass with proximal cancer and newly demonstrates the importance of adopting a broad definition of anaemia. We found that 80% of proximal cancer cases could be identified using the broad definition (with or without IDA), compared to 39% using the narrow definition with requirement for IDA. The broad definition should therefore be adopted when selecting patients for flexible sigmoidoscopy since there is a greater likelihood of missing proximal cancers with the narrower definition. Given the importance of anaemia as a marker for proximal cancer, we also recommend that all patients referred with suspected CRC have a full blood count, unless an emergency investigation is required.

Through closer examination of cancer yield by combinations of symptoms and signs, we were able to define the criteria for flexible sigmoidoscopy more specifically. Proximal cancer risk was particularly low in patients without anaemia and/or abdominal mass who presented with a CIBH to increased frequency alone. Similarly, very few proximal cancers were detected in patients without anaemia and/or abdominal mass who presented with rectal bleeding, alone or with other symptoms/signs. These novel findings led us to conclude that flexible sigmoidoscopy alone is sufficient for patients without .anaemia and/or abdominal mass who present with any rectal bleeding or solely a CIBH to increased frequency, unless there are significant distal findings that warrant WCI (i.e. large or multiple adenomas, IBD) or the examination is incomplete. Patients fitting these criteria could be reassured that their risk of proximal cancer is very low but that they should return to their GP if symptoms continue. In our study, 41% of patients fulfilled these criteria for flexible sigmoidoscopy alone.

The probability of missing a proximal cancer is an important consideration. In the study by Thompson et al., 131 proximal cancers were diagnosed.^8^ Of the 37 cases without anaemia and/or abdominal mass, 20 would have undergone subsequent WCI because of severe symptoms or significant flexible sigmoidoscopy findings. Thus 17 proximal cancers (13%) would have been missed if only flexible sigmoidoscopy was undertaken. With our criteria for flexible sigmoidoscopy, 10 proximal cancers (8%) would have been missed in the Thompson data, and probably fewer if our broad anaemia definition had been considered. Adoption of our flexible sigmoidoscopy criteria would therefore minimise the probability of missing proximal cancers.

There is a small risk that distal cancers could go undetected if flexible sigmoidoscopy is undertaken alone. While flexible sigmoidoscopy, in theory, can reach the splenic flexure, the descending colon sometimes goes unexamined as there are no anatomical markers that the splenic flexure has been reached.^20,23^ Few cancers are, however, located in the descending colon.^24^ In our study, 8 of the 429 distal cancers were in the descending colon and only 1 of the 8 patients would have undergone flexible sigmoidoscopy alone with our criteria.

There is also a small risk of missing cancers in the sigmoid colon with flexible sigmoidoscopy. Examinations failing to reach the sigmoid–descending colon junction are, however, typically deemed incomplete,^20,23,25^ prompting subsequent whole-colon completion examination.^8,9,21^ It is worth noting that, although studies have reported high rates of incomplete examinations due to pain, faeces, or scope looping, these were based on flexible sigmoidoscopy performed over 10 years ago.^23,25^ There are now remedies to avoid these scenarios. Premixed 50% nitrous oxide and oxygen (Entonox, BOC Healthcare, Guildford, Surrey, UK) has been shown to be as effective as conventional sedation for colonoscopy and is used frequently for flexible sigmoidoscopy screening in the English Bowel Cancer Screening programme.^26^ Preparation for flexible sigmoidoscopy usually involves a single phosphate enema,^6^ although oral preparations are occasionally used. If the distal bowel is not adequately cleared by an initial enema, a second can be administered via the endoscope. This has proved highly effective in patients having inadequate bowel preparation at colonoscopy.^27^

Isolated proximal non-cancerous abnormalities could also be missed by undertaking flexible sigmoidoscopy alone. Few studies, however, have addressed this.^28,29^ One retrospective study of 1766 patients who had undergone colonoscopy for rectal bleeding found isolated proximal colitis in 26 (1.5%) patients.^28^ Similarly, in a study of 625 patients aged <50 years with diarrhoea, 10 (1.6%) had isolated proximal IBD or colitis.^29^ Microscopic colitis, a common cause of unexplained chronic diarrhoea, presents a particular challenge as it can only be diagnosed on colonic biopsy and the vast majority of patients who receive a diagnosis have macroscopically normal colons.^30^ Although limited, the current evidence indicates that, when microscopic colitis is present, it is present throughout the colon.^31,32^ It is therefore likely that distal biopsies taken during flexible sigmoidoscopy would be sufficient for diagnosis.

Our study has several strengths, including recruitment from multiple hospitals, detailed baseline patient information, and follow-up using cancer registries for 3 years post-referral. We are confident that the follow-up period was sufficiently long to capture all incident CRC cases as the vast majority of CRCs were diagnosed within 6 months, with very few diagnosed in the ensuing 3 years.

Limitations include a possible selection bias since all patients were aged ≥55 years, considered fit for WCI, and 72% were urgently referred. Some patients did not proceed to undergo WCI but we were unable to accurately identify these patients from hospital records; nevertheless, we are confident that we did not miss cancers because we had cancer registry data for 3 years post-referral. Reporting bias is possible as the recording of symptoms by medical staff may have been influenced by what investigations were planned or by the presumed significance of the symptoms.^33^

A further limitation is that we did not have blood count data on all patients; for those patients without this data, anaemia was defined by whether it was indicated as a reason for referral. With full blood count data, it is possible that even fewer proximal cancer cases would have been missed by our flexible sigmoidoscopy criteria, as a greater proportion may have had anaemia. Finally, it is important to note that the cohort was gathered before the Bowel Cancer Screening Programme was fully rolled out in England. It is now common in clinical practice to consider the screening history of patients along with how recently any colonic examinations have taken place when selecting the most appropriate diagnostic investigation.

## Conclusion

Our findings confirm the strong association between anaemia and proximal cancer, with 80% of proximal cancer patients in our cohort meeting the criteria for broad definition anaemia. All patients with suspected CRC should therefore have a full blood count, unless an emergency investigation is needed, and patients identified as anaemic according to the broad definition of anaemia should be referred for WCI. Conversely, the risk of proximal cancer is very low in patients without broad definition anaemia and/or abdominal mass but who present with any rectal bleeding or solely a CIBH to increased frequency. Patients with this symptom profile can be examined safely by flexible sigmoidoscopy alone. If incorporated into guidelines, this strategy would greatly reduce the number of WCIs performed in patients at low risk of proximal cancer; this would alleviate the burden of WCI on patients and endoscopy and radiology services.

## Electronic supplementary material

Supplementary Tables

## Availability of data and materials

Because of the sensitive nature of the data collected for this study, requests to access the data set from qualified researchers trained in human subject confidentiality protocols may be sent to the corresponding author.
