# Supplementary material for: Whole-colon investigation vs. flexible sigmoidoscopy for suspected colorectal cancer based on presenting symptoms and signs: a multicentre cohort study
Source: Br J Cancer. 2018 Dec 19;120(2):154–64. doi: 10.1038/s41416-018-0335-z (PMC6342953; doi:10.1038/s41416-018-0335-z)
Supplement: Supplementary file 1 — Supplementary Tables [file 41416_2018_335_MOESM1_ESM.docx]

**Supplementary Table 1: Site of cancer diagnoses (N=7375)**

| **Site of cancer** | **N (%)** | **Diagnostic yield - %** | **Proportion of all patients with cancer - %** |
| --- | --- | --- | --- |
| **All sites** | 551* | 7.5 | 100 |
| **Distal cancers** |  |  |  |
| Total patients with distal cancer | 429* | 5.8 | 77.9 |
| Anus | 10 | 0.1 | 1.8 |
| Rectum | 210 | 2.8 | 38.1 |
| Rectosigmoid | 57 | 0.8 | 10.3 |
| Sigmoid colon | 146 | 2.0 | 26.5 |
| Descending colon | 8 | 0.1 | 1.5 |
| Distal colon (no further specification) | 4 | 0.1 | 0.7 |
| **Proximal cancers** |  |  |  |
| Total patients with proximal cancer | 127* | 1.7 | 23.0 |
| Splenic flexure | 9 | 0.1 | 1.6 |
| Transverse colon | 18 | 0.2 | 3.3 |
| Hepatic flexure | 14 | 0.2 | 2.5 |
| Ascending colon | 36 | 0.5 | 6.5 |
| Caecum | 53 | 0.7 | 9.6 |

* Five patients were diagnosed with synchronous distal and proximal cancers.

**Supplementary Table 2: Patient characteristics according to availability of blood count data**

| **Characteristic** | **Total cohort (N=7375)** | | **Cohort with blood count data (N=4742)*** | | **Cohort without blood count data (N=2633)** | | ***p*-value†** |
| --- | --- | --- | --- | --- | --- | --- | --- |
| **Sex** | **n** | **%** | **n** | **%** | **n** | **%** |  |
| Men | 3023 | 41·0 | 1,950 | 41.1 | 1,073 | 40.8 | 0.76 |
| Women | 4352 | 59·0 | 2,792 | 58.9 | 1,560 | 59.2 |  |
| **Age (years)** |  |  |  |  |  |  |  |
| 55-64 | 2407 | 32·6 | 1,418 | 29.9 | 989 | 37.6 | <0.0001 |
| 65-74 | 2739 | 37·1 | 1,800 | 38.0 | 939 | 35.7 |  |
| 75-84 | 1896 | 25·7 | 1,289 | 27.2 | 607 | 23.0 |  |
| ≥85 | 333 | 4·5 | 235 | 5.0 | 98 | 3.7 |  |
| **Route of referral** |  |  |  |  |  |  |  |
| Colorectal surgical outpatient clinic | 6231 | 84·5 | 3,848 | 81.1 | 2,383 | 90.5 | <0.0001 |
| Other outpatient clinic | 688 | 9·3 | 537 | 11.3 | 151 | 5.7 |  |
| Straight to test | 396 | 5·4 | 309 | 6.5 | 87 | 3.3 |  |
| Hospital admission | 33 | 0·4 | 26 | 0.5 | 7 | 0.3 |  |
| Not recorded | 27 | 0·4 | 22 | 0.5 | 5 | 0.2 |  |
| **Urgency of referral** |  |  |  |  |  |  |  |
| Urgent | 5290 | 71·7 | 3,393 | 71.6 | 1,897 | 72.0 | 0.0003 |
| Soon | 660 | 9·0 | 410 | 8.6 | 250 | 9.5 |  |
| Routine | 914 | 12·4 | 568 | 12.0 | 346 | 13.1 |  |
| Not recorded | 511 | 6·9 | 371 | 7.8 | 140 | 5.3 |  |

*The cohort with blood count data (N=4742) all had haemoglobin (Hb) and mean red cell corpuscular volume (MCV) counts. There were 1157 patients with serum ferritin in addition to Hb and MCV counts.

†*p*-values calculated with the χ² test to compare the cohort with blood count data to the cohort without blood count data.

**Supplementary Table 3: Number of additional NICE 2015 guideline symptoms or signs per patient by symptom or sign**

|  | **Total patients** | **Number of additional NICE 2015 guideline symptoms or signs per patient*** | | | | | | | |  |
| --- | --- | --- | --- | --- | --- | --- | --- | --- | --- | --- |
|  |  | **None** | | **1** | | **2** | | **3+** | | |
|  | **n** | **n** | **%** | **n** | **%** | **n** | **%** | **n** | **%** | |
| **Total** | 7375 | 2884 | 39.1 | 2958 | 40.1 | 1243 | 16.9 | 290 | 3.9 | |
| **Symptoms** |  |  |  |  |  |  |  |  |  | |
| Change in bowel habit | 5382 | 1589 | 29.5 | 2394 | 44.5 | 1121 | 20.8 | 278 | 5.2 | |
| More frequent | 2862 | 908 | 31.7 | 1255 | 43.9 | 561 | 19.6 | 138 | 4.8 | |
| Less frequent | 865 | 189 | 21.8 | 399 | 46.1 | 224 | 25.9 | 53 | 6.1 | |
| Variable | 648 | 183 | 28.2 | 307 | 47.4 | 126 | 19.4 | 32 | 4.9 | |
| Unspecified | 1007 | 309 | 30.7 | 433 | 43.0 | 210 | 20.9 | 55 | 5.5 | |
| Rectal bleeding | 2773 | 618 | 22.3 | 1257 | 45.3 | 696 | 25.1 | 202 | 7.3 | |
| Abdominal pain | 2126 | 192 | 9.0 | 1033 | 48.6 | 692 | 32.5 | 209 | 9.8 | |
| Weight loss | 1148 | 18 | 1.6 | 433 | 37.7 | 488 | 42.5 | 209 | 18.2 | |
| Other symptoms^†^ | 479 | 13 | 2.7 | 143 | 29.9 | 187 | 39.0 | 136 | 28.4 | |
| **Signs/indications** |  |  |  |  |  |  |  |  |  | |
| Anaemia^‡^ | 1889 | 404 | 21.4 | 670 | 35.5 | 595 | 31.5 | 220 | 11.6 | |
| Abdominal mass | 216 | 12 | 5.6 | 68 | 31.5 | 78 | 36.1 | 58 | 26.9 | |
| Rectal mass | 165 | 19 | 11.5 | 62 | 37.6 | 59 | 35.8 | 25 | 15.2 | |
| Other signs^§^ | 265 | 20 | 7.5 | 120 | 45.3 | 89 | 33.6 | 36 | 13.6 | |

* NICE 2015 guideline symptoms/signs included change in bowel habit (CIBH), rectal bleeding, abdominal pain, weight loss, anaemia and abdominal or rectal mass without any restriction by age; the broad definition of anaemia was included. Patients may have had multiple symptoms/signs.

† Other symptoms include bloating/flatulence (n=203); tiredness/weakness (n=152); anal symptoms (n=97); nausea/vomiting (n=44); back pain (n=13); and upper gastrointestinal symptoms (n=10).

‡ In patients with blood count data, anaemia was defined by the WHO (broad) definition [haemoglobin (Hb) level <13g/dL in men or <12g/dL in women]. In patients without blood count data, anaemia was defined by whether the investigation of anaemia was indicated as a reason for referral.

§ Other signs include faecal occult blood test (FOBT) positivity (n=113); family history (n=117); history of polyps (n=23); cancer antibodies (n=3); elevated C-reactive protein (n=4); and liver problems (n=9).

**Supplementary Table 4: Proximal cancers in patients without anaemia or abdominal mass presenting with any rectal bleeding and/or a change in bowel habit to more frequent stools alone**

| **Patient** | **Sex** | **Age (years)** | **Cancer site** | **Cancer size (mm)** | **Haemoglobin result** | **Date of Hb test relative to trial registration** | **Symptoms and signs** | **Distal findings** |
| --- | --- | --- | --- | --- | --- | --- | --- | --- |
| **Distal findings that would necessitate referral for whole colon exam** | | | | | | | | |
| 1 | Male | 80 | CM | Unknown | NA | - | RB, CIBH (more frequent), AP | FS finding of suspected cancer in RM |
| 2 | Female | 59 | AC | 45 | NA | - | RB, CIBH (more frequent) | 20mm tubulovillous adenoma in SC |
| 3 | Female | 69 | CM | 50 | 13.4 g/dL | 18 days prior | RB | Transported blood observed distally; multiple sessile polyps in RM (<4mm) |
| 4 | Female | 77 | CM | Unknown | NA | - | RB | FS finding of >3 lesions |
| 5 | Female | 82 | HF | 35 | 12.1 g/dL | 30 days prior | RB, CIBH (more frequent), AP, WL | Synchronous 48mm distal cancer in SC |
| 6 | Male | 63 | SF | 35 | 15.7 g/dL | 0 days | RB | 8mm tubulovillous adenoma in RM |
| **No reason for whole colon exam** | | | | | | | | |
| 7 | Male | 69 | TC | 35 | NA | - | RB, CIBH (more frequent) | Multiple diverticula |
| 8 | Male | 71 | TC | 25 | NA | - | RB, CIBH (more frequent) | 3 hyperplastic polyps in SC (all ≤7mm), multiple diverticula |
| 9 | Male | 79 | AC | 30 | NA | - | RB, CIBH (more frequent) | 5mm tubular adenoma in SC, proctitis in RM |
| 10 | Female | 65 | CM | 70 | 12.0 g/dL | 0 days | RB, CIBH more frequent) | Multiple diverticula |
| 11 | Female | 83 | CM | 70 | NA | - | RB, CIBH (more frequent) | No reported abnormality |
| **No information on findings known** | | | | | | | | |
| 12 | Male | 81 | CM | 30 | NA | - | RB, CIBH (more frequent) | No abnormality reported |
| 13 | Female | 68 | CM | Unknown | 12.5 g/dL | 43 days prior | RB, CIBH (more frequent) | No information |

Abbreviations: AC, ascending colon; AP, abdominal pain; CIBH, change in bowel habit; CM, caecum; FS, flexible sigmoidoscopy; HF, hepatic flexure; NA, not available; RB, rectal bleeding; RM, rectum; SC, sigmoid colon; SF, splenic flexure; TC, transverse colon; WL, weight loss.

**Supplementary Table 5: Symptoms and signs among patients with descending colon cancers**

| **Patient** | **Sex** | **Age (years)** | **Cancer size (mm)** | **Haemoglobin (Hb) result** | **Date of Hb test relative to trial registration** | **Symptoms and signs** | **Other distal findings** |
| --- | --- | --- | --- | --- | --- | --- | --- |
| 1 | Male | 59 | 70 | 12.7 | 5 days prior | Anaemia, WL, AP | Minor diverticular change in SC |
| 2 | Female | 79 | 70 | 9.4 | 0 days | Anaemia, AM, WL, AP CIBH (unspecified), weakness | Moderate diverticular disease in SC |
| 3 | Male | 70 | 230 | 10.7 | 1 day after | Anaemia, WL, CIBH (unspecified), weakness | No other abnormality reported |
| 4 | Male | 60 | 60 | 15.8 | 0 days | AM, AP, CIBH (unspecified), bloating | Up to 20 smaller polyps in RM & SC (hyperplastic polyps and mildly dysplastic tubular adenomas) |
| 5 | Female | 58 | 35 | 14.3 | 53 days prior | CIBH (more frequent), FOBT positive | Severe diverticular disease in SC |
| 6 | Male | 78 | Unknown | NA | - | CIBH (unspecified) | 15 mm polyp in SC and diverticular disease in SC; synchronous proximal cancer in TC |
| 7 | Male | 63 | 50 | NA | - | RB, CIBH(more frequent) | 50mm distal cancer in SC |
| 8 | Male | 72 | 11 | 13.1 | 76 days prior | RB, CIBH(more frequent) | No other abnormality reported |

Abbreviations: WL, weight loss; AP, abdominal pain; SC, sigmoid colon; AM, abdominal mass; CIBH, change in bowel habit; RM, rectum; FOBT, faecal occult blood test; NA, not available; TC, transverse colon; RB, rectal bleeding.
